# Supplementary material for: The interactome of intact mitochondria by cross-linking mass spectrometry provides evidence for coexisting respiratory supercomplexes
Source: Mol Cell Proteomics. 2017 Dec 8;17(2):216–32. doi: 10.1074/mcp.RA117.000470 (PMC5795388; doi:10.1074/mcp.RA117.000470)
Supplement: Supplemental Data [file supp_RA117.000470_133922_0_supp_23978_fzffwf.zip › spectra_annotation/mito_DR_spectra_annotation/464-1-7-1-5-1.pdf]

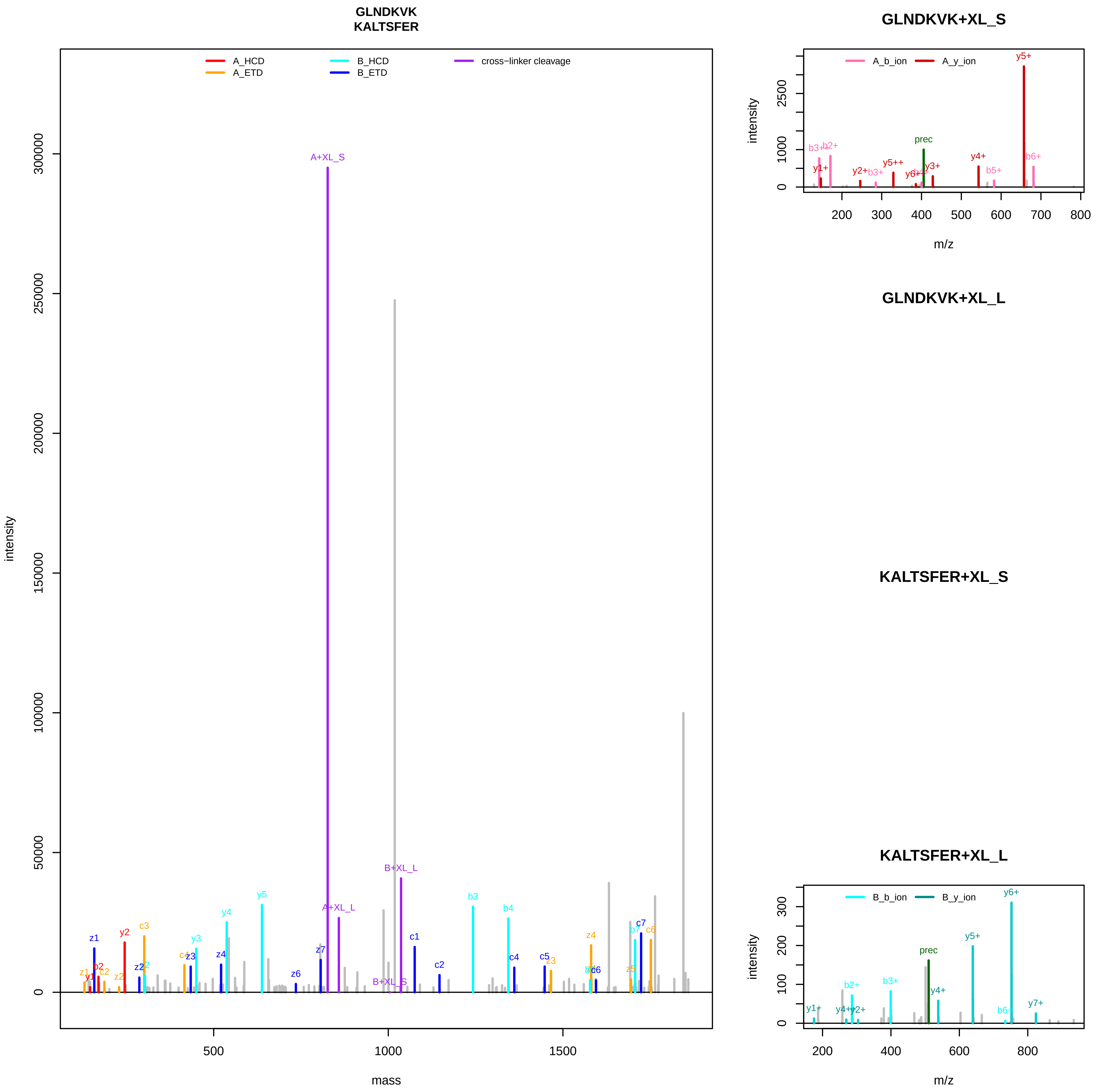

GLNDKVK
KALTSFER
A_HCD
A_ETD
B_HCD
B_ETD
cross−linker cleavage
A+XL_S
A+XL_L
B+XL_L
B+XL_S
intensity
mass
0
50000
100000
150000
200000
250000
300000
500
1000
1500
GLNDKVK+XL_S
A_b_ion
A_y_ion
prec
y5+
y4+
b6+
b5+
y3+
y5++
y2+
y1+
b3++
b2+
b3+
m/z
200
300
400
500
600
700
800
1000
2500
GLNDKVK+XL_L
KALTSFER+XL_S
KALTSFER+XL_L
B_b_ion
B_y_ion
y6+
y5+
y4+
y7+
b2+
b3+
y1+
b6+
100
200
300
